# Development of Flexible and Conductive Immiscible Thermoplastic/Elastomer Monofilament for Smart Textiles Applications Using 3D Printing

**DOI:** 10.3390/polym12102300

**Published:** 2020-10-08

**Authors:** Prisca Aude Eutionnat-Diffo, Aurélie Cayla, Yan Chen, Jinping Guan, Vincent Nierstrasz, Christine Campagne

**Affiliations:** 1 ENSAIT, GEMTEX–Laboratoire de Génie et Matériaux Textiles, F-59000 Lille, France; aurelie.cayla@ensait.fr (A.C.); christine.campagne@ensait.fr (C.C.); 2 Textile Materials Technology, Department of Textile Technology, Faculty of Textiles, Engineering and Business, University of Borås, 50190 Borås, Sweden; vincent.nierstrasz@hb.se; 3 College of Textile and Clothing Engineering, Soochow University, Suzhou 2150060, Jiangsu, China; yanchen@suda.edu.cn (Y.C.); guanjinping@suda.edu.cn (J.G.)

**Keywords:** 3D printing, conductive polymer composite (CPC), immiscible polymer blends, co-continuity, location of fillers, deformation, stress and strain, electrical conductivity

## Abstract

3D printing utilized as a direct deposition of conductive polymeric materials onto textiles reveals to be an attractive technique in the development of functional textiles. However, the conductive fillers—filled thermoplastic polymers commonly used in the development of functional textiles through 3D printing technology and most specifically through Fused Deposition Modeling (FDM) process—are not appropriate for textile applications as they are excessively brittle and fragile at room temperature. Indeed, a large amount of fillers is incorporated into the polymers to attain the percolation threshold increasing their viscosity and stiffness. For this reason, this study focuses on enhancing the flexibility, stress and strain at rupture and electrical conductivity of 3D-printed conductive polymer onto textiles by developing various immiscible polymer blends. A phase is composed of a conductive polymer composite (CPC) made of a carbon nanotubes (CNT) and highly structured carbon black (KB)- filled low-density polyethylene (LDPE) and another one of propylene-based elastomer (PBE) blends. Two requirements are essential to create flexible and highly conductive monofilaments for 3D-printed polymers onto textile materials applications. First, the co-continuity of both the thermoplastic and the elastomer phases and the location of the conductive fillers in the thermoplastic phase or at the interface of the two immiscible polymers are necessary to preserve the flexibility of the elastomer while decreasing the global amount of charges in the blends. In the present work based on theoretical models, when using a two-step melt process, the KB and CNT particles are found to be both preferentially located at the LDPE/PBE interface. Moreover, in the case of the two-step extrusion, SEM characterization showed that the KB particles were located in the LDPE while the CNT were mainly at the LDPE/PBE interface and TEM analysis demonstrated that KB and CNT nanoparticles were in LDPE and at the interface. For one-step extrusion, it was found that both KB and CNT are in the PBE and LDPE phases. These selective locations play a key role in extending the co-continuity of the LDPE and PBE phases over a much larger composition range. Therefore, the melt flow index and the electrical conductivity of monofilament, the deformation under compression, the strain and stress and the electrical conductivity of the 3D-printed conducting polymer composite onto textiles were significantly improved with KB and CNT-filled LDPE/PBE blends compared to KB and CNT-filled LDPE separately. The two-step extrusion processed $60\%({\mathrm{LDPE}}_{16.7\%\;\mathrm{KB}\;+\;4.2\%\;\mathrm{CNT}})/40\;\mathrm{PBE}$ blends presented the best properties and almost similar to the ones of the textile materials and henceforth, could be a better material for functional textile development through 3D printing onto textiles.

## 1. Introduction

Fused Deposition Modeling (FDM), a 3D printing technique, is used to deposit fused conductive thermoplastic polymers onto textile materials, following, for instance, a desired pattern and a defined number of layers. This technology has drawn the attention of several researchers in the field of functional textiles as it allows the integration of sensors, antennas and conductive tracks on the surface of the textile materials [1,2,3,4,5,6,7,8,9,10,11]. However, the added functional elements to the surface of the textiles should confer same or better properties than the ones of the original fabrics. 

The most important properties are the deformations, the tensile properties, the abrasion resistance, the washability and the adhesion between the textile and the printed track. The adhesion between the 3D-printed layers and the textile has already been investigated by a few researchers [2,3,4,8,10,12]. They have tried to understand and optimize it through different methods, for instance, the selection of thermoplastic polymer, textile characteristics and definition of the optimal 3D printing parameters. In addition, Eutionnat-Diffo et al. [3] studied the stress, strain and deformation under pressure of the 3D-printed polylactic acid (PLA) and carbon-black (CB)-filled PLA onto textiles. They found that the deposition process significantly influences the tensile and deformation properties of the printed polymer onto textile compared to the ones of the textiles [4]. The stress and strain at rupture of the 3D-printed PLA and CB-filled PLA layers are much lower than the one of the polyethylene terephthalate (PET) fabric. Consequently, an improvement of the strain and stress of the conductive and non-conductive layer printed onto the fabrics is needed. Besides, the deformations under constant pressure of the 3D-printed PLA on PET fabrics are lower than the ones of the fabric, i.e., the materials after 3D printing are more rigid and it breaks rapidly after bending. However, to the best of the authors’ knowledge, no researchers have suggested more flexible and elastic polymer blends for 3D printing onto textile’s surface applications while controlling the electrical properties. 

High-density polyethylene (HDPE) is a polyolefin thermoplastic already used in 3D printing by Schirmeister et al. [13]. Despite the existing challenges (shrinkage, voiding and warpage) in the 3D printing of HDPE using Fused Deposition Modeling (FDM), they could define appropriate 3D printing parameters, such as nozzle temperature and extrusion rate, to obtain HDPE parts with improved tensile strength and Young’s modulus [13]. However, softer thermoplastic polymers than the commonly used in 3D printing, such as the acrylonitrile butadiene styrene (ABS) and PLA, still need to be explored. Low-density polyethylene (LDPE) is defined as a polymer polymerized from ethylene which has a highly branched structure composed of both long and short branches that interfere with crystallization. Its very low glass transition and lower percent crystallinity make it softer and more flexible, exhibiting rubbery state at ambient temperature compared to HDPE and other thermoplastic polymers [14]. Besides, polyethylene (PE) is widely used in the textiles field. As a result, LDPE is an alternative polymer that can be used in the 3D printing onto textiles field. Additionally, conducting polymer composites (CPCs) are popularly used in the development of smart elements such as electrodes, sensors and antennas [15]. CPCs are defined as electrically conducting nanoparticle-filled insulating polymeric matrices above the percolation threshold. The sensing mechanism of CPCs is based on the polymer’s reaction to environmental changes which affect the electrically conductive CNT network. For instance, the variation of temperature of the environment leads to an increase or decrease of the CPC resistance [15,16,17,18]. Generally, added fillers into polymer increase the viscosity and stiffness of the CPCs and decrease its 3D printing processability [7,16,19,20]. The viscosity, the stiffness and the processability can be enhanced by blending the CPCs with an immiscible elastomeric polymer. With their properties varying from plastic to elastomer, polyolefins are the most widely used polymers [21]. Due to their low density, better chemical resistance, low cost and good resilience without permanent deformation, the polyolefin-based elastomers have recently received considerable attention. Their fabrication process does not require vulcanization. Therefore, Exxon has developed the propylene-based ethylene (PBE) elastomers using metallocene catalysts by the Exxpol™ technology. In the case of flexible monofilament development, both polymers should be immiscible to create preferably two co-continuous phases where the conductive fillers will be only located into the CPCs or at the interface. In immiscible polymer blends, the morphology of the two phases and the properties of the component polymers determine the performance of the blends [22]. In general, depending on their contents in the blend, when one polymer is dispersed in another polymer, a sea-island microstructure (also called nodular structure) is formed. By increasing the content of the minor component up to a critical value, the morphology changes from a nodular or fibrillar structure into a co-continuous one. It was found that the addition of nanoparticles into immiscible polymer blends extends the phase co-continuity over a much larger composition range [23,24,25,26,27,28]. The morphology and electrical properties of carbon black (CB)-filled polystyrene (PS)/polyethylene (PE) blends were approached by Gubbels et al. [29]. They found that the addition of 4 wt% of CB was selectively located in the PE phase, which maintained the co-continuous structure with 40 wt% and 10 wt% of PE. Similar effects could also be obtained with carbon nanotube-filled immiscible polymer [30]. The location of the nanofillers is important as it determines the morphology and formation of the network of nanoparticles in the structure. Cayla et al. [19] studied two immiscible blend systems of polycaprolactone (PCL) with polypropylene (PP) and polyamide 12 (PA12) with CNTs. After introducing the CNTs in the PCL phase, the CPC was blended with the PP or PA12. Despite the use of a similar mixing sequence, CNT location was different in the two blends. Indeed, it was proven that the CNTs remained in the PCL phase while it migrated from PCL to PA12. Furthermore, Marischal et al. [19] examined the morphology of three immiscible blends at various ratios: CNT-filled PCL/PP, CNT-filled PCL/ polyamide 6 (PA6) and CNT-filled PCL/polyethylene terephthalate (PET) and the location of the CNTs. Based on their findings, they conclude that with a co-continuous structure and selective location of the CNTs in the polycaprolactone, the blend (CNT-filled PCL)50%/PP50% is the most appropriate for polymer-based textile heating systems.

The main target of this research work is to enhance the flexibility, stress and strain at rupture and electrical conductivity of 3D-printed conductive polymer onto textiles by producing immiscible carbon nanotubes (CNT) and highly structured carbon black known as Ketjenblack (KB)-filled low-density polyethylene (LDPE)/propylene-based elastomer (PBE) blends. The rheological and electrical properties of the CPCs/PBE blends, as well as the prediction of the location of the fillers in the various studied blends, were investigated. In addition, deformation under compression, strain and stress and the electrical conductivity of the 3D-printed CPCs/PBE blends onto textiles were explored. 

In this study, the appellation “3D-PPOT conductive materials” means 3D-printed conductive polymers on textiles materials.

## 2. Materials and Methods 

### 2.1. Materials

A 14-picks/inch twill woven fabric made of PET twisted multi-filaments of Nm 40 as warp yarn and polyester monofilament of 0.2 mm in diameter as weft yarn was used for this study. Nm is defined as the number of hanks of 1000 meters/kg. Low-density polyethylene of 0.918 g/cm^3^ density [31] (ref: 25-15G150453) and the propylene-based elastomer granulates of 0.861 g/cm^3^ [32] density (ref: Vistamaxx 6202) were purchased from Dow Chemical, Horgen, Switzerland and ExxonMobil Chemical, Texas, USA, respectively. Vistamaxx 6202 is an ethylene/propylene random copolymer produced by ExxonMobil′s metallocene catalyst technique. X. Wang et al. demonstrated that the whole molecular chain of propylene-based elastomer consisted of soft segments at room temperature [33]. 

Highly structured carbon black named Ketjenblack (KB) (ref: EC600JD) and multiwalled carbon nanotubes (MWCNT) (ref: NC 7000) were supplied by Nouryon, Amsterdam, Netherlands and Nanocyl, Sambreville, Belgium respectively. These MWCNTs have an average length of approximately 1.5 µm, a diameter of 10 nm, and a specific area of 250 m^2^/g. The Ketjenblack nanoparticles have a high surface area of 1400 m^2^/g.

### 2.2. Method

#### 2.2.1. Design of Experiments

The samples A1 to A14 of the first design of experiments are described in Table 1. The first design was approached in order to have a general understanding on the influence of each factor on the properties of the conductive LDPE/PBE blends and the 3D-PPOT materials. As it can be seen in Table 1, the weight percentages of fillers introduced in the LDPE are not the same as the ones after blending them with PBE. Indeed, the total weight percentages of the fillers in the conducting polymer composites (CPCs)/PBE blends were different because a dilution of the fillers occurred when the ratio of PBE in the blend was increased. For example, the samples A3, A7 and A11 have 10, 6 and 4 wt% KB in the blends respectively. This decrease in weight percentages might lead to a decrease in the electrical conductivity properties of the conductive monofilament and the 3D-PPOT materials. For each run of samples, the morphological, rheological and electrical properties of (CPCs)/PBE blends were explored with four replicates. 

A second design of experiments using five different samples B1 to B5, presented in Table 2, was used to study both the properties (morphological, rheological and electrical) of the CPCs and the polymer blends presented in Table 2 and the properties of the 3D-PPOT materials (deformation, tensile and electrical) using the developed CPCs. In the second experiment, the weight percentages of the fillers contained in the CPCs/PBE blends was the targeted one (wt% KB = 10 and wt% CNT = 2.5) and the same for each blend after extrusion. Indeed, a higher amount of fillers, calculated based on the weight percentage of LDPE-based CPCs, was introduced in the LDPE (Table 2). 

#### 2.2.2. Preparation of the Oompounds 

**Figure 1:**
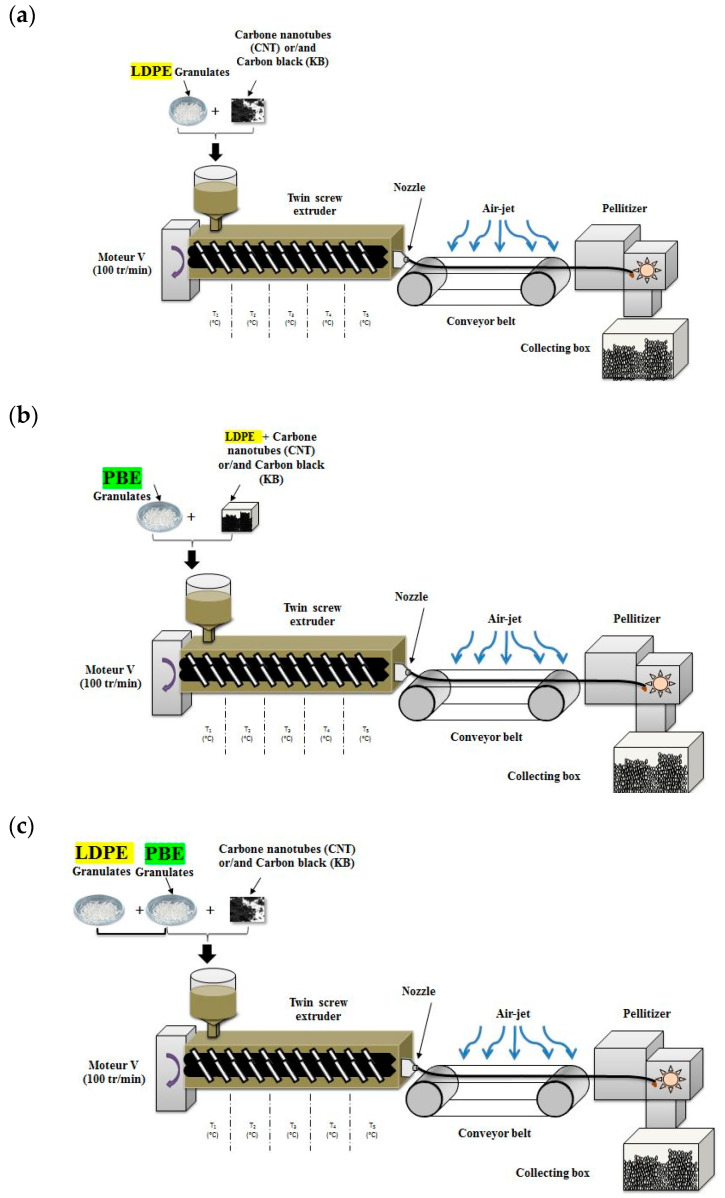
Scheme of the 2-step extrusion (**a**,**b**) and 1-step extrusion (**c**). The first step of the two-step extrusion is presented in (**a**) and the second step in (**b**).

Each extrusion process was executed using a co-rotating intermeshing twin-screw extruder (ref: PTW 16/25p with length/diameter = 25) from Thermo-Haake with a rotating speed of 100 RPM and a shear stress close to 20 s^−1^. Prior to extrusion, the LDPE and PBE granulates were dried at 70 °C and 40 °C, respectively, for 12 h. Two extrusion scenarios are approached in the second design of experiments described in Section 2.2.1. The first one, named 1-step extrusion (Figure 1c), consists of blending of the LDPE, PBE and fillers (MWCNT and/or KB) in one step by homogeneously dispersing the fillers in the polymer blends. LDPE and PBE were used in the form of granulates. The 2-step extrusion is accomplished in two stages (Figure 1a,b): a first dispersion of KB and CNT in the LDPE granulates and extrusion of the CPCs followed by a second extrusion of LDPE-based CPC with PBE at different percentages according to a specific design of experiments detailed in the following Section 2.2.1. Before the second extrusion, the LDPE-based CPC monofilaments were converted into granulates. Finally, after the second extrusion, the filler-filled biphasic blend monofilaments were converted into granulates in order to be processed in the 3D printer. Both scenarios were investigated in order to understand their impact on the selective location of fillers in the biphasic CPC/PBE blends after extrusion. 

The temperature profiles, used for the five zones of the extruder for each blend, are presented in Table 3. 

Specific appellation was used in Table 3 to define the polymer blends. For example, $60{\mathrm{LDPE}}_{\mathrm{KB}\;10\;\mathrm{CNT}\;5}/40\mathrm{PBE}$ describes 60% of 10% KB and 5% CNT–filled LDPE, blended with 40% of PBE. Similar designations are utilized in the entire article. 

#### 2.2.3. Rheological Measurements 

The Melt Flow Index (MFI) measures the ease of melt flow of polymeric material, i.e., the flow-ability of the polymer at a certain temperature in limited time of 10 min. The melt flow tester from ThermoHaake was used in this study, according to the standard ISO-11333. For the first experiment (samples A1–A14), the test was executed at 200 °C and 2.16 kg (standard conditions of the MFI test defined in ISO-11333), whereas for the second experiment (samples B1–B5) it was done at 245 °C and 10 kg (cf. Section 2.2.1 for description of experiments). The second experiment was executed at 245 °C because at 200 °C, the KB and CNT-filled LDPE/PBE blends could not go through the nozzle during the test. The samples B1–B5 are more viscous than the samples A1–14 due to much higher KB and CNT contents creating percolation networks. At a certain weight percentage of fillers, the movement of the macromolecular chains of the polymer or polymer blend is very limited. An increase of the weight and temperature was necessary to be able to evaluate and compare the samples of the second experiment.

The test consists of various steps. During the first step, the piston is placed inside the chamber of the machine at a set temperature for 4 min and then, the polymer is loaded and pre-heated at the same temperature during 4 min to finally be pushed through the die. Every 15 s, a knife was used to automatically cut the extruded polymer. The polymer is then collected and weighted to obtain the MFI value. Prior to the test, the material was dried at 40 °C for 12 h. Five replicates were performed in this test and the MFI value was given in g/10 min. 

#### 2.2.4. 3D Printing Process

**Figure 2:**
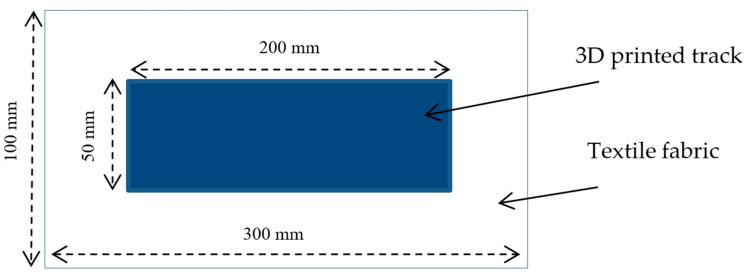
Scheme of the polymer 3D-printed onto textiles for mechanical tests.

Polyester woven samples of rectangular shape (100 mm × 300 mm) were placed directly on the metallic build platform of the Pellet Additive Manufacturing (PAM) printer from Pollen (France) based at Euromaterials (France) prior to the printing process. Then, a thin and rectangular layer (50 mm × 200 mm × 0.1 mm) made of LDPE-based CPCs and LDPE-based CPCs/PBE blends, designed first on Rhinoceros CAD software and then imported into Simplify 3D software, was printed on each different set of woven fabrics. The rectangular layer was needed to realize the mechanical (tensile and deformation) tests on the samples (Figure 2). A square layer (50 mm × 50 mm × 0.1 mm) was designed for electrical conductivity measurements. The printing parameters are presented in Table 4. The distance between the head of the extruder and the surface of the textile was set during the calibration and remained constant and only the same extruder was used for all the different trials.

#### 2.2.5. Filler Location Prediction Based on Contact Angle and Wettability Coefficient Determination

The contact angle between one-millimeter polymer films made of 100% PBE and two different liquids (water and α-bromonaphthalene) with specific and different surface tensions was determined through a GBX MCAT V6 Digidrop (Dublin, Ireland). Prior to measurement, films of PBE were prepared through thermocompression. The theoretical values of the surface tensions for these two liquids are presented in Table 5 [20]. The contact angles were measured after the 30 µL drop of wetting liquids was standing for 30 s at room temperature. Five drops of each liquid were used to obtain an average value of the contact angle. 

Through various equations already demonstrated by Fowkes [34] and Cardinaud et al. [35], the interfacial energies between the particles and the polymer were calculated with the contact angle values. The surface tension, its dispersive and polar components of the LDPE, KB and CNT nanoparticles used in the calculation were found in the literature [19,36,37]. Finally, the location of the fillers in each biphasic blend was determined by using Equation (1).

(1) $$\omega_{A-B}=\frac{\gamma_{\mathrm{filler}\;1-B}-\gamma_{\mathrm{filler}\;1-A}}{\gamma_{A-B}}$$

where ω_A−B_ is the wettability coefficient between the polymers A and B, γ_filler 1-B_ is the interfacial energy between filler 1 and polymer B (Nm/m) and γ_A-B_ is the interfacial energy between polymer A and polymer B. 

Several cases can occur:

- The wettability coefficient is lower than −1, which means that filler 1 is located in polymer B;
- The wettability coefficient is between −1 and 1, which means that filler 1 is found at the interface of the two polymers;
- The wettability coefficient is higher than 1, which means that filler 1 is in polymer A.

#### 2.2.6. Thickness Measurement

A thickness gauge, micrometer KES-FB3 was utilized to measure the thickness of each fabric according to the standard ISO 5084.

#### 2.2.7. Stress and Strain at Break

The stress and strain at break measurements were executed on 3D-PPOT (3D-Printed conductive polymers on textiles) materials following the standard ISO 13934-1. The speed of each experiment was 100 mm/min and the dimensions of the samples were 150 and 25 mm, respectively. The distance between the two clamps was 50 mm. An average of three measurements was done and the accepted standard deviation was 10%.

#### 2.2.8. Morphology Analysis through Scanning Electron Microscopy (SEM)

Samples were immerged in liquid nitrogen for the duration of 3 minutes before being cut in longitudinal and transverse directions by the cryogenic fracture of each monofilament. Then, they were chrome- or carbon-metalized with a thickness of 300 Å. SEM images were obtained by means of an SEM Hitachi S4700 operating at different voltages (between 2 kV and 10 kV depending of the targets of the observations) and magnifications, at University of Lille in France. The different blends are made of immiscible polymers, therefore, two distinct phases are observed. As the LDPE is denser than the PBE elastomer (Section 2.1), the LDPE phase is whiter than the PBE phase. SEM visualizations of KB and CNT have already been revealed by researchers [38,39].

#### 2.2.9. Morphology Analysis through Transmission Electron Microscopy (TEM)

The equipment used was the TECNAI G^2^ operating at 200 kV and an exposure time of 0.60 s. Prior to the experiment, samples were cut at a temperature at −65 °C using a diamond knife to generate clean, flat surfaces in the longitudinal direction, and provide a clear picture of the polymer surface. Both PBE and LDPE phases are detectable through TEM as the brighter (lower density) and darker (higher density) phases. 

#### 2.2.10. Permanent, Elastic and Total Deformations 

Dynamic surface deformations (permanent, elastic and total) of the 3D-PPOT samples of the second design of experiments (Section 2.2.1) were determined by Universal Surface Tester (UST). They describe the flexibility behavior of these materials. The principle of the test was already described by Eutionnat-Diffo et al. [4] An average of four measurements were necessary to have an acceptable standard deviation. 

#### 2.2.11. Thermal Properties through Differential Scanning Calorimetry (DSC)

Differential scanning calorimeter (DSC) measurements were carried out with a DSC3 StarTM System (Mettler Toledo Swiss) under nitrogen atmosphere with a heating rate of 10 °C/min^−1^. The crystallization behavior and the degree of crystallization of conducting polymer composites and biphasic polymer blends, but also the influence of the PBE on the crystallization and melting behavior of LDPE are studied. During the DSC analysis, 6 mg samples were heated from 25  to 250 °C at 10 °C/min, then they were cooled down from 250 to 25 °C at 10 °C/min. This cycle is repeated twice with only the decrease of the first cycle and increase of the second cycle to remove the thermal history of the studied polymers CPCs and CPCs/PBE blends. The melting enthalpy and crystallinity of each component of the blends could not be determined as it was difficult to execute the deconvolution of the merged melting peaks. 

#### 2.2.12. Electrical Conductivity Measurement

The electrical conductivity measurement of the conducting monofilaments and the conducting 3D-PPOT materials was executed through a hand-made four-wire system [7] and a standardized Keithley 8009 Resistivity Test Fixture [40] box respectively according to ASTM D 257 standard, connected to a Keithley 2461 SourceMeter (Beaverton, OR, USA). Although a voltage value from −0.5 V to 3 V with an increment of 0.5 V was applied to the material, the current intensity was measured. The resistance was calculated using the slope of the characteristic curve. Finally, the electrical conductivities for monofilaments and 3D-PPOT materials were determined through Equations (2) and (3), respectively.

σ = L/(R × S)(2)

where σ is the electrical conductivity (S/m), R is the resistance of the sample (Ω) R = 1/slope, L is the distance between the electrodes (m), and S is the cross-sectional area of the sample (m^2^).

σ = 1/(R/S)(3)

where σ is the surface conductivity (S), R is the resistance of the sample (Ω) R = 53.4/slope and S is the cross-sectional area of the sample (m^2^).

The cross-sectional area of the monofilament sample was calculated after determining its diameter with an electronic caliper gauge. The range of the monofilament diameters was [1.2 mm; 2.1 mm]. Five measurements of the diameter were necessary to guarantee a standard deviation of 5%. For each monofilament, the diameter was homogenous within the filament length. 

## 3. Results

### 3.1. Analysis of Virgin LDPE/PBE Biphasic Polymer Blends

#### 3.1.1. DSC Analysis

**Figure 3:**
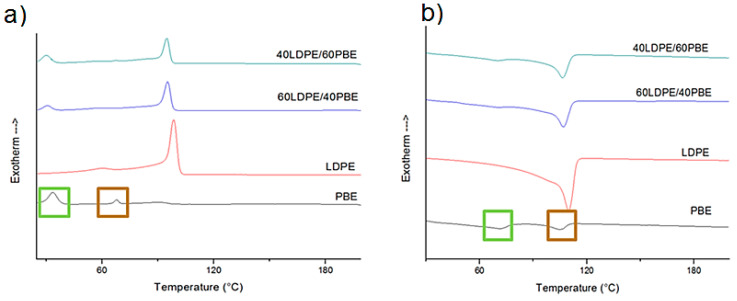
Differential scanning calorimetry (DSC) curves of PBE, LDPE and PBE/LDPE blends; (**a**) represents the crystallization curves and (**b**) the melting curves.

Figure 3 registers the DSC subsequent melting and crystallization curves of the PBE, the LDPE and LDPE/PBE blends. Two melting temperatures and two crystallization temperatures are noticeable on the melting curve of PBE. (b). Indeed, the PBE is a random propylene–ethylene copolymer which contains over 80% of propylene, and thus two distinct peaks should be observed. The peaks framed in green (T_c_ = 40 °C and T_c_ = 70 °C) and brown (T_m_ = 65 °C and T_c_ = 100 °C) are the ones of propylene and ethylene, respectively. Besides, one melting peak (T_m_ = 115 °C) and two crystallization peaks (T_c1_ = 110 °C and T_c2_ = 65 °C) were detected for LDPE. In the LDPE/PBE polymer blends, a merge of two melting peaks was visualized at 110 °C and another melting peak at 70 °C was recorded. Two crystallization peaks at 105 and 38 °C of the polymer blends are pictured. Thus, these observations demonstrated that, in the polymer blends, LDPE and PBE are two immiscible polymers.

#### 3.1.2. Rheology Analysis

**Figure 4:**
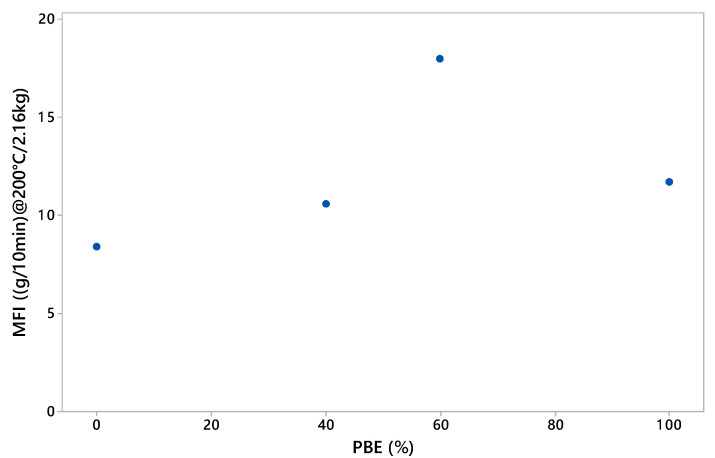
Melt Flow Index (MFI) (200 °C/2.16kg) values: effect of PBE percentage in LDPE/PBE blends.

The melt flow index (MFI) values of the biphasic LDPE/PBE blends are represented in Figure 4. The standard deviation of each value is very low; therefore, the error bars are not visible. The MFI value of the PBE (11.7 ± 0.1 g/10min) was revealed to be higher than the one of the LDPE (8.4 ± 0.2 g/10 min). However, by blending these two polymers, a rise in the MFI is observed while increasing the percentage of PBE in the blend. Moreover, the value of the LDPE_60_/PBE_40_ is higher than the one of the PBE. Ku and Lin already explained this phenomenon as an increase of the surface area of incompatible inter-phases of the polymer blends [41]. In our case, biphasic polymer blends were used and the surface area between the two phases was increased. This phenomenon is very rare but exists in some cases. Thus, the addition of PBE enhances considerably the processability of the CPCs through 3D printing.

#### 3.1.3. Morphology Analysis

**Figure 5:**
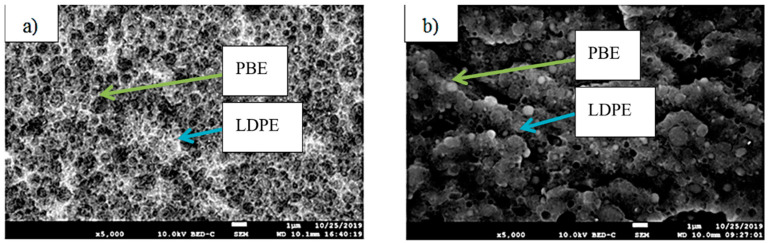
SEM images of the cross sections of 60%LDPE/40%PBE (**a**) and 40%LDPE/60%PBE (**b**). LDPE is whiter and in the PBE is darker.

The visualization of the morphologies is very important as it gives information on the final properties of the biphasic blends. LDPE is denser than PBE; consequently, on the SEM images presented in Figure 5, LDPE appears white and PBE dark grey. In both blends, there is a nodular dispersion of one phase in the other one. In other words, in 60%LDPE/40%PBE and 40%LDPE/60%PBE, nodules of PBE in LDPE and nodules of LDPE in PBE are noticed, respectively. Thus, those virgin blends can hardly be used to produce flexible 3D-PPOT materials through FDM as both phases (elastomeric and thermoplastic phases) need to be co-continuous to guarantee the flexibility of the entire 3D-printed track. The co-continuity of the conductive biphasic polymeric blends might be obtained depending on the selective location of the fillers. 

### 3.2. Analysis of Conducting LDPE/PBE Biphasic Polymer Blends

The location of the KB and CNT in the LDPE/PBE blends, the morphology, rheology and electrical properties and DSC analysis of the LDPE/PBE biphasic polymer blends are approached in this section. 

#### 3.2.1. Location of KB and CNT in LDPE/PBE Blends

a) Prediction of the Location through Theoretical Models

In order to calculate the wettability coefficient, the contact angles of PBE with water and α-Bromonaphtalene were measured and are listed in Table 6. Further, the interfacial energies (including dispersive and polar components) were calculated and are summarized in Table 7. Based on theoretical models using the wettability coefficient ($\omega_{A-B}$) already detailed by some researchers [19,20,42], the locations of KB and CNT were predicted prior to melt processing of the conducting LDPE/PBE blends. The wettability coefficient is defined in Equation (4). The locations were calculated with the surface tensions of the LDPE, PBE, CNT and KB. Both the wettability coefficient and the location of the fillers are presented in Table 8.

(4) $$\omega_{A-B}=\frac{\gamma_{\mathrm{filler}\;1-B}-\gamma_{\mathrm{filler}\;1-A}}{\gamma_{A-B}}$$

where ω_A−B_ is the wettability coefficient between the polymers A and B, γ_filler 1-B_ is the interfacial energy between filler 1 and polymer B (Nm/m) and γ_A-B_ is the interfacial energy between polymer A and polymer B. 

Several scenarios can happen:

1) The wettability coefficient is lower than −1 and the filler 1 is located in the polymer B;
2) The wettability coefficient is between −1 and 1 and the filler 1 is found at the interface of the two polymers;
3) The wettability coefficient is higher than 1 and the filler 1 is in the polymer A.

It was found that the wettability coefficient was between -1 and 1 for KB and CNT nanoparticles which are both located at the interface.

b) Location of Fillers through SEM Images

**Figure 6:**
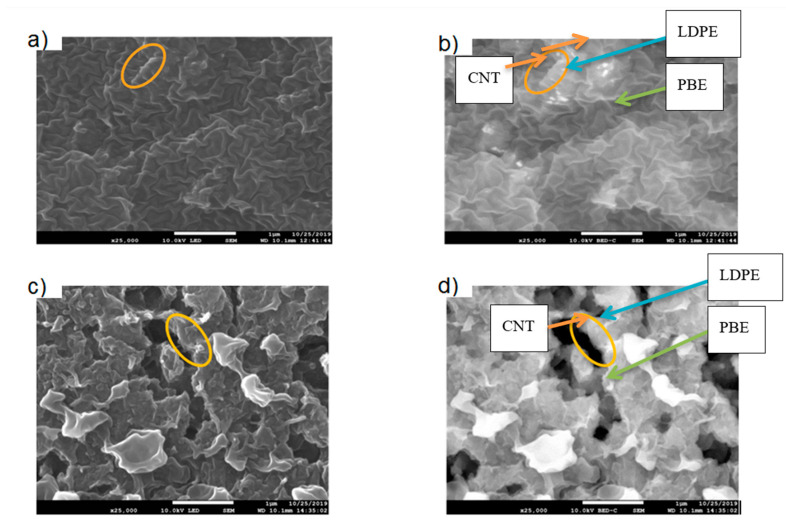
SEM images in cross section of the $40\left(LDPE_{KB\;10\;CNT\;5}\right)/60\;PBE$ and $60\left(LDPE_{KB\;10\;CNT\;5}\right)/40\;PBE$ blends: location of CNT nanoparticles. (**a**,**c**) are in normal LED mode and (**b**,**d**) are in backscattered mode. LDPE is whiter and PBE is darker.

**Figure 7:**
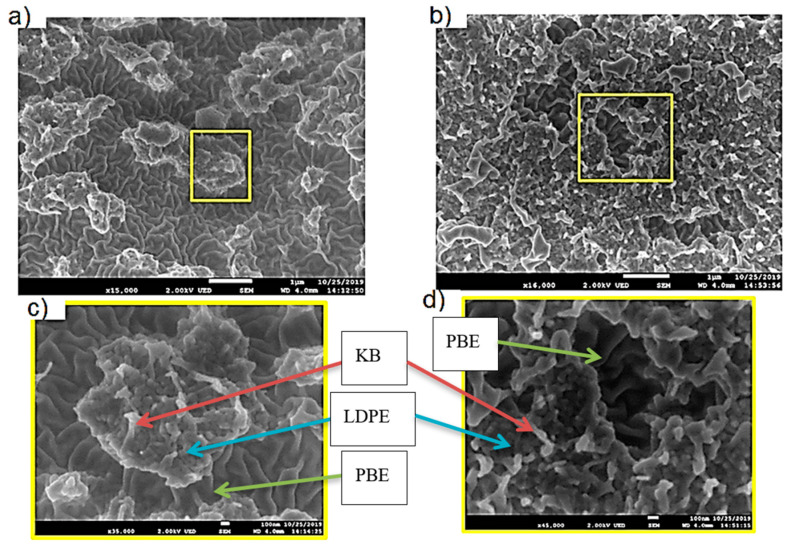
SEM images in cross section of the $40\left(LDPE_{KB\;10\;CNT\;5}\right)/60\;PBE$ (**a** and **c** which is the focus of **a**) and $60\left(LDPE_{KB\;10\;CNT\;5}\right)/40\;PBE$ (**b** and **d** which is the focus of **b**) blends: location of KB nanoparticles. LDPE is whiter and PBE is darker.

The location of KB and CNT nanoparticles in the $40\left({\mathrm{LDPE}}_{\mathrm{KB}\;10\;\mathrm{CNT}\;5}\right)/60\;\mathrm{PBE}$ and $60\left({\mathrm{LDPE}}_{\mathrm{KB}\;10\;\mathrm{CNT}\;5}\right)/40\;\mathrm{PBE}$ blends was partially confirmed through the SEM images shown in Figure 5 and Figure 6. Indeed, CNT nanoparticles seem to be located only at the interface and the KBs in the LDPE and at the interface. As the propylene-based elastomer is only composed of soft segment, a difference in morphology between the PBE and the LDPE could be visualized through SEM images (Figure 6 and Figure 7). In all the blends, there were no KB nanoparticles detected in the PBE. 

#### 3.2.2. Morphology Analysis

**Figure 8:**
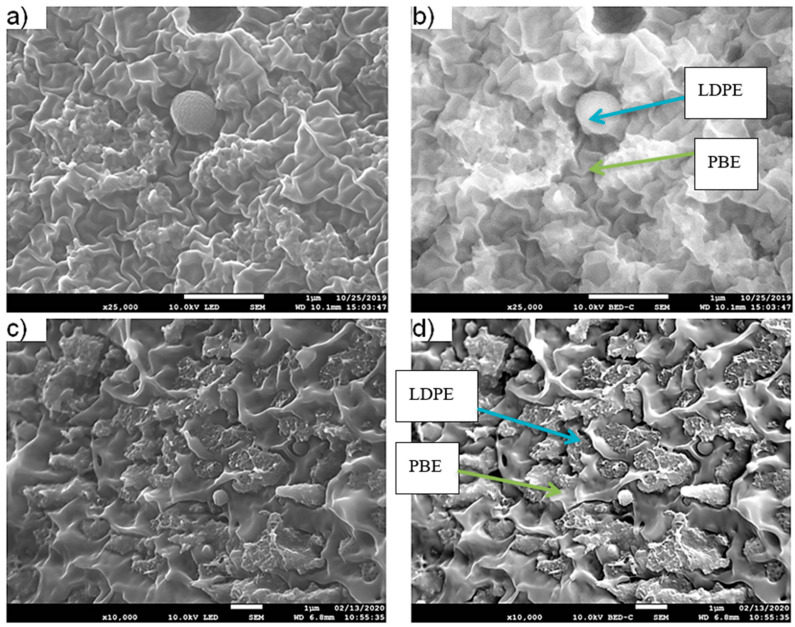
SEM images of the $40\left(LDPE_{KB\;10}\right)/60PBE$ blend in the transverse (**a**,**b**) and longitudinal directions (**c**,**d**). (**a**,**c**) are in normal LED mode and (**b**) and (**d**) are in backscattered mode. LDPE is whiter and the PBE is darker.

The morphology of the $40\left({\mathrm{LDPE}}_{\mathrm{KB}\;10}\right)/60\mathrm{PBE}$ blend is shown in Figure 8. In the transverse direction, two distinct phases (PBE and LDPE) were detected. However, some nodules of LDPE were observed in the PBE phase. In the longitudinal direction, a fibrillar structure with LDPE and PBE phases was perceived. Similar morphology was obtained with the $40\left({\mathrm{LDPE}}_{\mathrm{KB}\;5}\right)/60\mathrm{PBE}$ blend. Thus, the addition of maximum 10% of KB particles in the LDPE prior to dispersion in PBE did not allow the structure to be co-continuous. 

**Figure 9:**
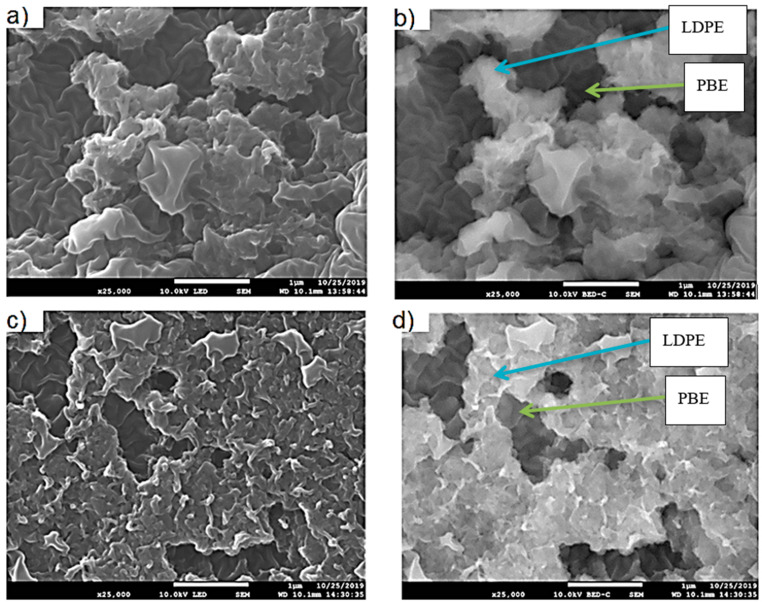
SEM images of the $40\left(LDPE_{KB\;10\;CNT\;5}\right)/60PBE$ (**a**,**b**) and $60\left(LDPE_{KB\;10\;CNT\;5}\right)/40PBE$ (**c**,**d**) in the transverse directions. (**a**,**c**) are in normal LED mode and (**b**,**d**) are in backscattered mode. LDPE is whiter and the PBE is darker.

However, as shown in Figure 9, the co-continuity of both phases is observed when introducing both CNT and KB in the LDPE prior to mixing it with the PBE. A. Nuzzo et al. investigated the mechanisms of nanoparticle-induced co-continuity in immiscible polymer blends [43]. They found that orgaoclay, sepiolite and carbon nanotube-filled Polylactic acid (PLA)/Polyamide 11 (PA11) blends presented co-continuous phases as the three fillers are preferentially located in the minor PA phase allowing the initial drop-matrix morphology being a highly co-continuous one. Furthermore, the assembly of percolating filler networks, the ability of the nanoparticles to lower the interfacial tension between the two phases and the deformability of the nanoparticles induced the co-continuity of both phases [43]. In our case, in all the conducting biphasic blends using KB and CNT, the KB and CNT nanoparticles were preferentially located at the interface and in the LDPE and only at the interface, respectively. Thus, the CNTs and KBs might have changed the interfacial tension between the two phases. Additionally, the KB has extended the phase of LDPE from nodular structure with KB to co-continuous phase with KB. The results are observed for both blends of polymer nanocomposites $40\left({\mathrm{LDPE}}_{\mathrm{KB}\;10\;\mathrm{CNT}\;5}\right)/60\mathrm{PBE}$ and $60\left({\mathrm{LDPE}}_{\mathrm{KB}\;10}\right)/40\mathrm{PBE}$.

#### 3.2.3. Rheology Analysis 

**Figure 10:**
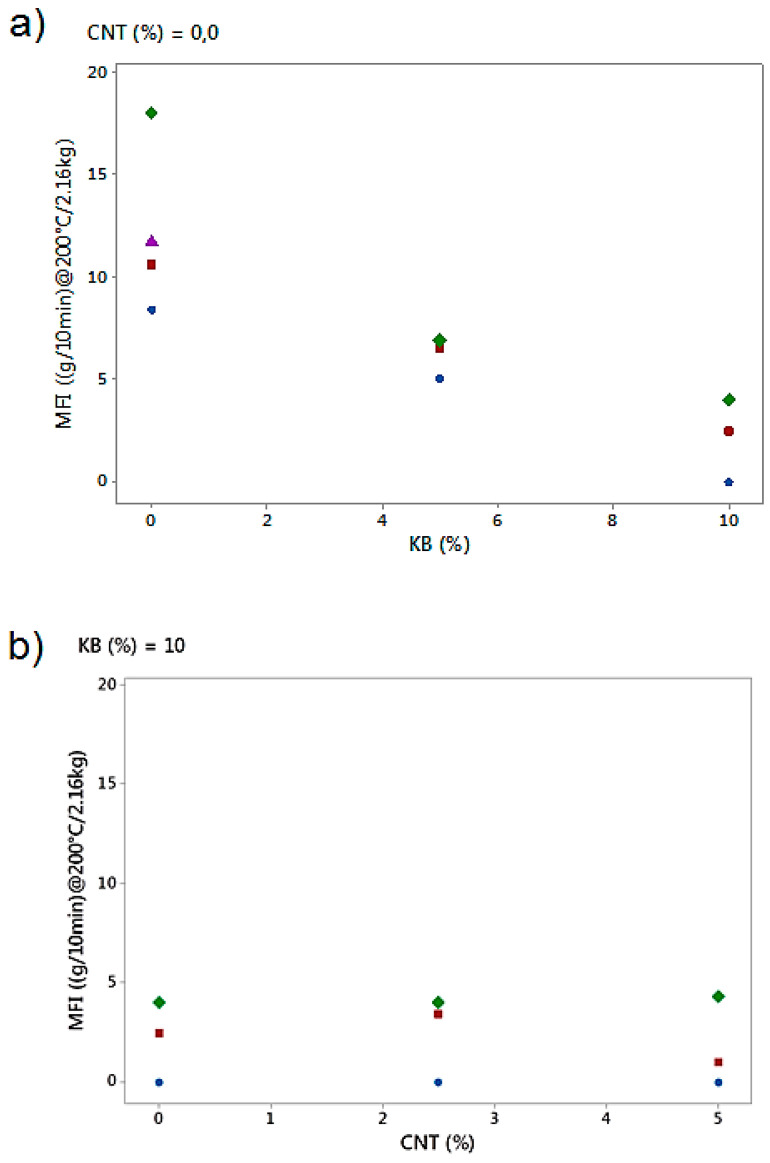
Effect of percentages of KB (**a**) and CNT (**b**) on the MFI (g/min at 200 °C/2.16 kg) of the conducting biphasic blends depending on the percentage of PBE. The blends containing 0%, 40%, 60% and 100% of PBE are represented in blue, red, green and purple.

The effect of KB and CNT percentages on the rheology properties through MFI analysis was also investigated (Figure 10). It was found that the increase of the amount of KB in the blends tends to decrease the Melt Flow Index. By increasing the percentage of KB in the biphasic blends, more networks are created between the fillers, reducing the mobility of the macromolecular chains of the LDPE and, thus, of all biphasic polymer blends. Moreover, the addition of CNT fillers did not change the MFI of the conducting biphasic blends. This might be due to the amount of KB already being very high (10%) while varying the amount the CNT fillers. 

#### 3.2.4. Differential Scanning Calorimetry (DSC) Analysis

**Figure 11:**
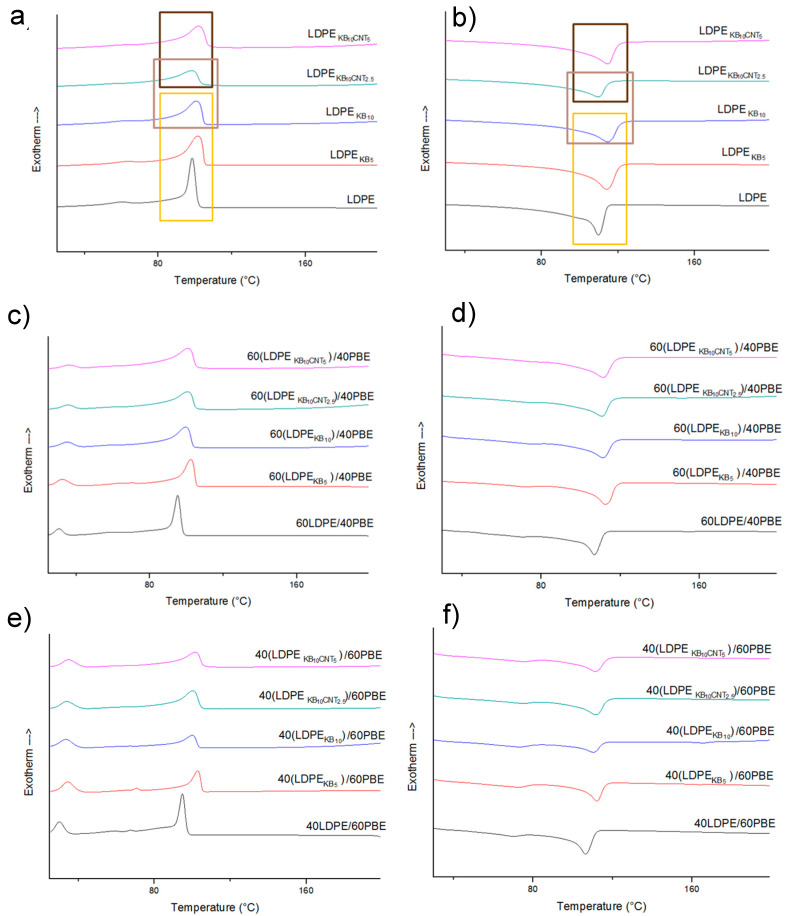
Crystallization (**a**,**c**,**e**) and melting (**b**,**d**,**f**) curves of the conducting biphasic polymers using different ratios of PBE, KB and CNT fillers.

**Figure 12:**
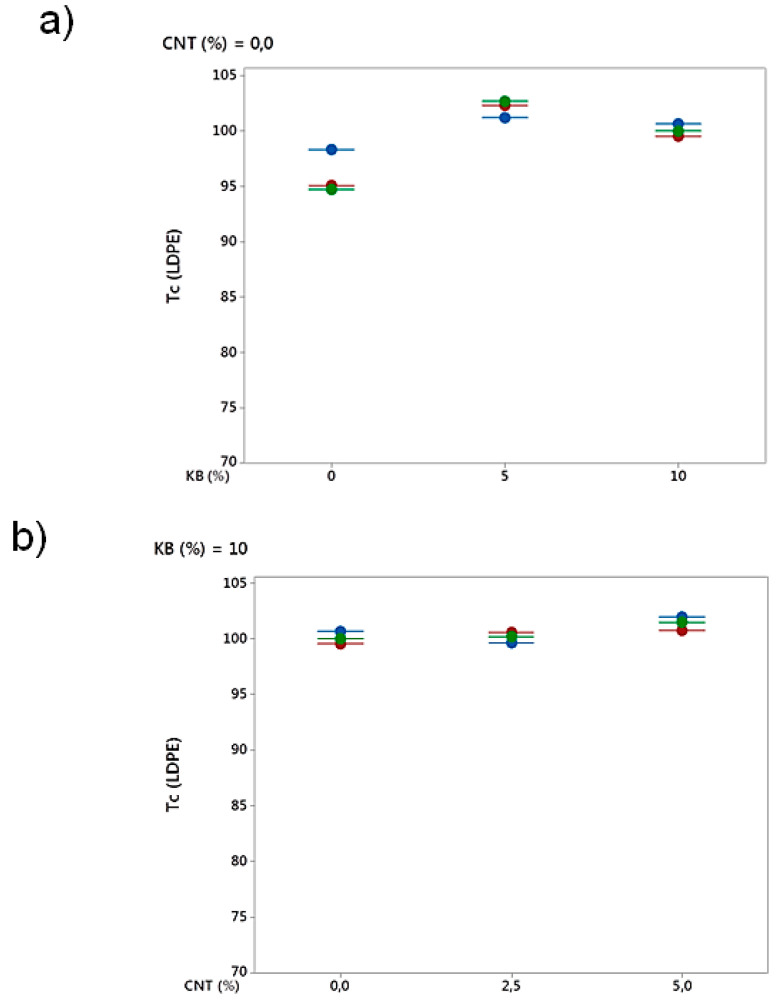
Effect of percentages of KB (**a**) and CNT (**b**) on the $T_{c}$ (°C) of the conducting biphasic LDPE-based CPC/PBE blends. The blends 100%LDPE-based CPC/0%PBE, 60% LDPE-based CPC/ 40%PBE and 40% LDPE-based CPC/60%PBE are represented in blue, red and green, respectively.

The effect of CNT and KB fillers on crystallinity behavior of the conducting blends was also approached and the melting and crystallization curves are presented in Figure 11. It was shown that KB and CNT fillers influence the crystallization of LDPE and PBE. An increase of the percentage of CNT trends to shift the crystallization temperatures to higher temperatures due to the nucleation effect of the CNT located at the interface [44,45,46,47]. Similar trends of findings were obtained for all the blends (Figure 11). Besides, adding more KB particles quadratically affected the crystallization temperature. (Figure 12). The CNT nanoparticles did not significantly influence the crystallization temperature. 

#### 3.2.5. Electrical Conductivity of Conducting LDPE/PBE Biphasic Polymer Blends in Monofilament

**Figure 13:**
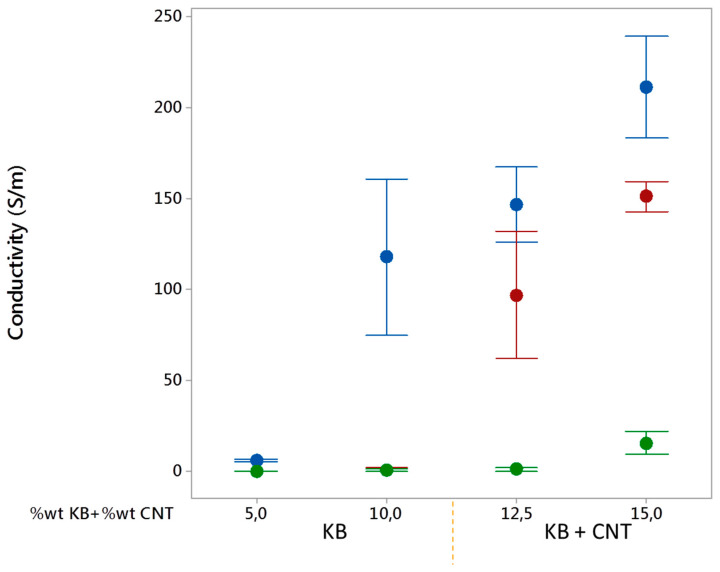
Electrical conductivity (S/m) as a function of KB and CNT ratios in the KB and CNT-filled LDPE. The CPCs without PBE are presented in blue, the 60 wt%CPC/40 wt% PBE blends in red and the 40 wt%CPCs/60 wt%PBE blends in green.

The electrical conductivity of the conductive biphasic polymeric blends is an important property to study for them to be applicable in smart textiles through 3D printing techniques. The need for developing biphasic polymers with co-continuous phases is justified by an enhancement of the electrical conductivity while decreasing the percolation threshold with a low amount of fillers in the blends [20,43]. The increase of the KB and CNT percentages led to increasing the electrical conductivity of the conducting polymers (Figure 13). It can be explained by the creation of inter-networks between the conductive fillers (synergistic effect). Furthermore, by blending the conducting LDPE with the PBE, the electrical conductivity diminished due to the dilution of the fillers in the blends. The more added PBE, the lower the electrical conductivity. The very low conductivity of the blends containing only KB filers can be explained by the nodular or fibrillar structure of these blends (Figure 8). 

**Figure 14:**
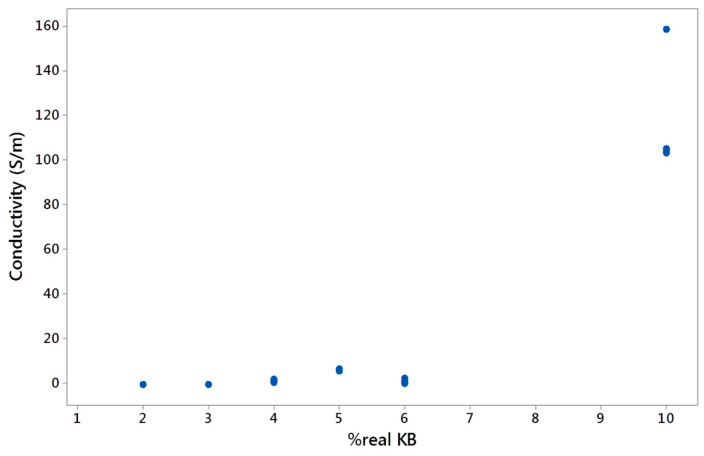
Electrical conductivity (S/m) as a function of the real KB percentage in the biphasic blends.

**Figure 15:**
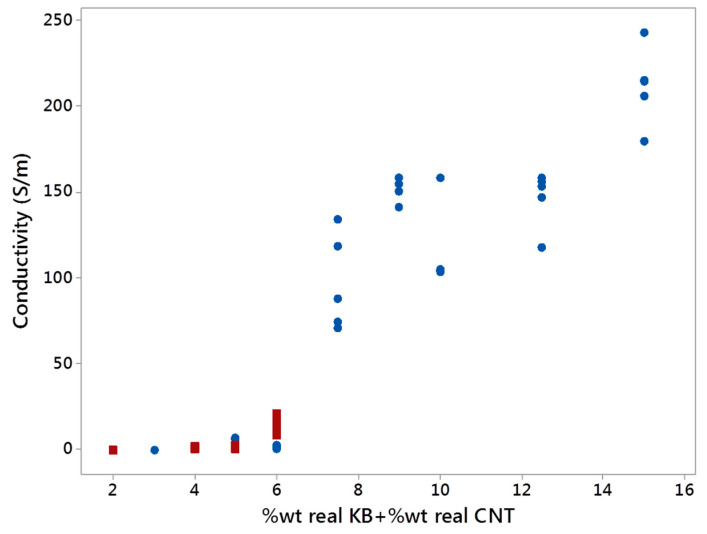
Electrical conductivity (S/m) as a function of the real KB and CNT percentages in the biphasic blends with a dominance of LDPE (in blue) and dominance of PBE (in red).

The amount and dispersion of the KB and CNT fillers in the LDPE highly influence the electrical conductivity of the final LDPE-based CPC. Indeed, at certain filler contents, the CPC might have reached the percolation threshold and become conductive. However, once the CPC is blended with PBE, the percolation threshold could change due to a different dispersion and distribution of the fillers in a larger volume of the biphasic polymers and also the modification of the morphology of the biphasic blends. A double percolation threshold occurs: a percolation of fillers and another one of CPC phases. Thus, the fillers are diluted in the blends and higher contents of fillers (CNT and KB) are necessary to reach the percolation threshold. In other words, the electrical conductivity depends on the global (or real) percentage of KB and CNT in the blends (Figure 14 and Figure 15). By mixing conducting LDPE with PBE at the different ratios, the global KB and CNT percentages in the blend decreased drastically and, therefore, reduced considerably the electrical conductivity of the blends (Figure 15). 

### 3.3. Analysis of Conducting LDPE/PBE Biphasic Polymer Blends at An Equivalent Global Ratio of Fillers

Previously, it has been demonstrated that the electrical properties of the LDPE-based CPC/PBE blends decreases with an increase of the PBE content because the fillers are diluted. As a result, novel conducting LDPE/PBE blends at similar filler ratios were developed and characterized. The targeted global CNT and KB ratios were 2.5wt% and 10wt% respectively. In other words, depending on the weight percentage of CNT and KB-filled LDPE and PBE added to the CNT and KB-filled LDPE/PBE blends, the quantity of CNT and KB (in wt%) incorporated into the LDPE at the first step was determined in order to obtain final weight percentages of CNT and KB equal to 2.5wt% and 10wt%, respectively. Thus, the filler ratios in the polymer blends were constant for any blends. The details of the amount of CNT and KB added in LDPE are presented in Table 2. 

#### 3.3.1. Morphology Analysis 

**Figure 16:**
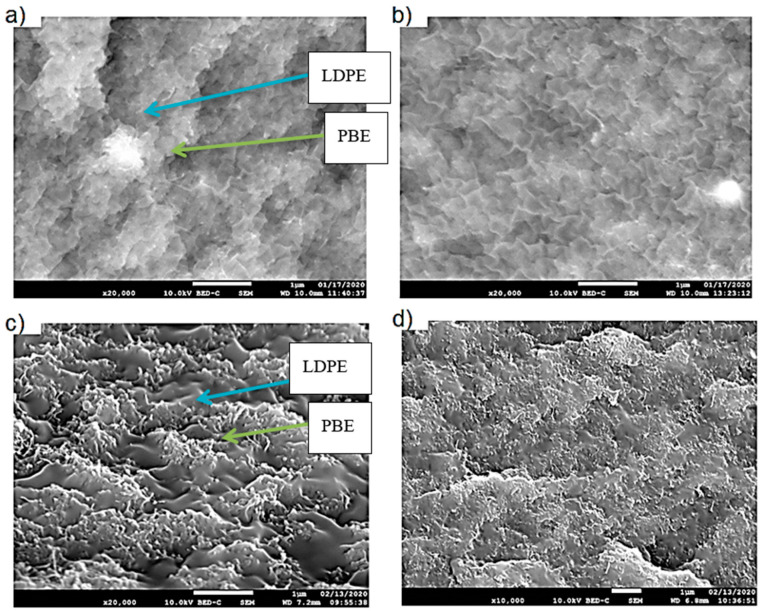
SEM images of the $60\left(LDPE_{KB\;16.7\;CNT\;4.2}\right)/40PBE$ to obtain globally in the blend 10% KB and 2.5% CNT. (**a**,**b**) and (**c**,**d**) represent the cross section and longitudinal views, respectively, of the blend using one-step and two-step extrusions. LDPE is whiter and the PBE is darker.

**Figure 17:**
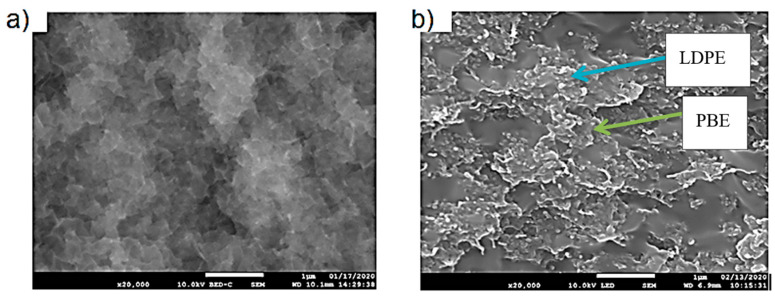
SEM images of the $80\left(LDPE_{KB\;12.5\;CNT\;3.1}\right)/20PBE$ to obtain globally in the blend 10% KB and 2.5% CNT. The images (**a**,**b**) represent the cross section and longitudinal views, respectively, of the blend using two-step extrusions. LDPE is whiter and the PBE is darker.

The morphology of the blends $60\left({\mathrm{LDPE}}_{\mathrm{KB}\;16.7\;\mathrm{CNT}\;4.2}\right)/40\mathrm{PBE}$ and $80\left({\mathrm{LDPE}}_{\mathrm{KB}\;12.5\;\mathrm{CNT}\;3.1}\right)/20\mathrm{PBE}\;$is represented by SEM images (Figure 16 and Figure 17). It could be noticed that the structure of both phases (LDPE and PBE) is co-continuous. However, due to an important amount of fillers, theoretically present at the interface or in the LDPE phase, a clear distinction between the phases can hardly be perceived. Indeed, the minor phase seems to be diluted into the other one and this effect seems to happen when mixing all the fillers in the PBE and LDPE granulates during one step.

#### 3.3.2. Location of Fillers 

**Figure 18:**
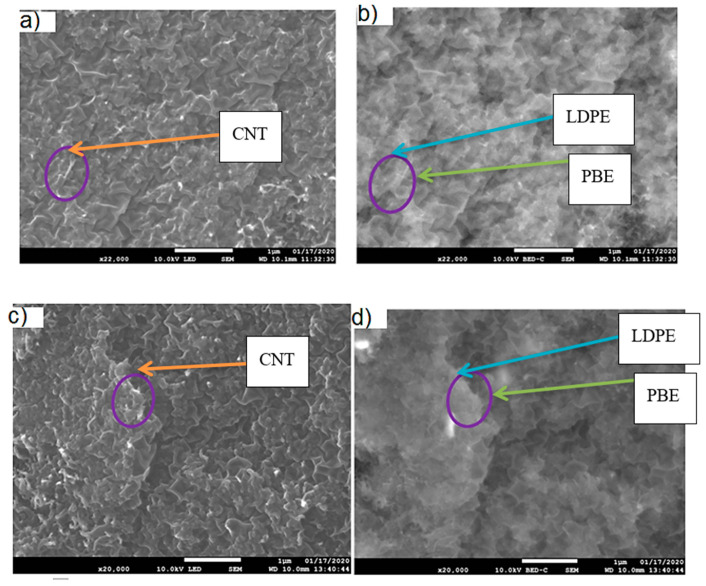
SEM images in the transverse direction of the one-step (**a**,**b**) and two-step (**c**,**d**) $60\left(LDPE_{KB\;16.7\;CNT\;4.2}\right)/40PBE$ blend: location of the CNT nanoparticles. (**a**) and (**c**) are in the normal LED mode and (**b**) and (**d**) are in backscattered mode. LDPE is whiter and the PBE is darker.

**Figure 19:**
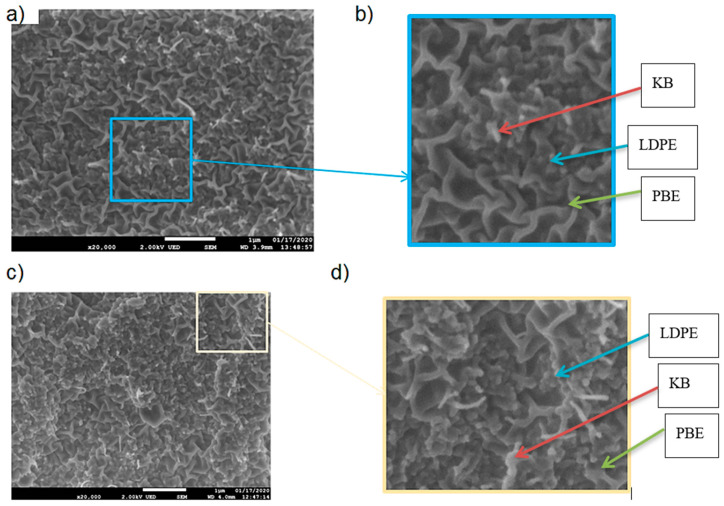
SEM images in the transverse direction of the 1-step (**a**,**b**) and 2-step (**c**,**d**) extruded $60\left(LDPE_{KB\;16.7\;CNT\;4.2}\right)/40PBE$ blend: location of the KB nanoparticles. LDPE is whiter and the PBE is darker.

**Figure 20:**
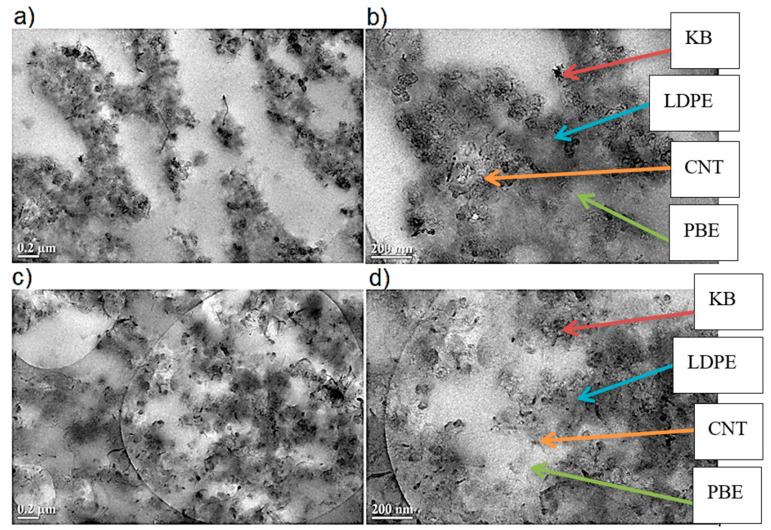
TEM images in the transverse direction of the 2-step (**a**,**b**) and 1-step (**c**,**d**) extruded $60\left(LDPE_{KB\;16.7\;CNT\;4.2}\right)/40PBE$ blend: location of the KB and CNT nanoparticles. LDPE is darker and the PBE is whiter.

The location of KB and CNT nanoparticles in the $60\left({\mathrm{LDPE}}_{\mathrm{KB}\;10\;\mathrm{CNT}\;5}\right)/40\;\mathrm{PBE}$ blend, prepared using one- and two-step extrusion, was observed through SEM images shown in Figure 18 and Figure 19. Indeed, CNT and KB nanoparticles seem to be located at the interface and/or in the LDPE (Figure 18) and not in the soft segments of PBE (Figure 19). These observations were also confirmed by TEM measurements and the results are presented in Figure 20. In the case of two-step extruded $60\left({\mathrm{LDPE}}_{\mathrm{KB}\;16.7\;\mathrm{CNT}\;4.2}\right)/40\mathrm{PBE}$, it can be perceived that the KB and the CNT nanoparticles were located in the LDPE and at the interface LDPE/PBE and no nanoparticles were in the PBE phase (Figure 11a,b). Whereas, for the 1-step extruded $60\left({\mathrm{LDPE}}_{\mathrm{KB}\;16.7\;\mathrm{CNT}\;4.2}\right)/40\mathrm{PBE}$ blends, CNT nanoparticles were visualized in both the LDPE and PBE phases and KB in LDPE (Figure 20c,d).

#### 3.3.3. Rheology Analysis 

**Figure 21:**
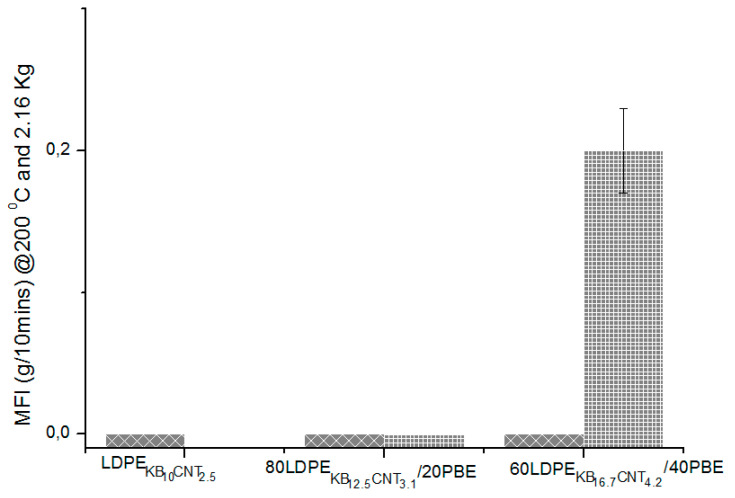
MFI (g/min @ 200 °C/2.16 kg) of the conducting biphasic blends using 1-step (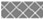) or 2-step (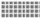) extrusion.

**Figure 22:**
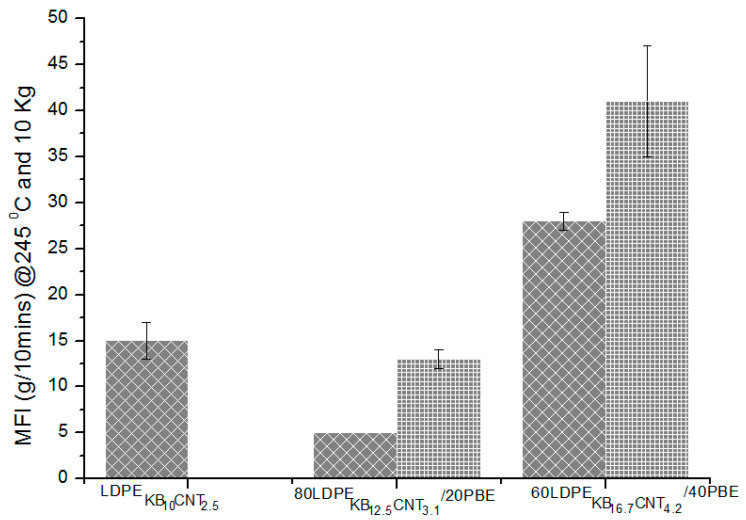
MFI (g/min @ 245 °C/10Kg) of the conducting biphasic blends using 1-step (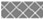)or 2-step (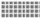) extrusion.

The values of the melt flow indexes at 245 °C/10 kg and 200 °C/2.16 kg of the nanocomposites blends are shown in Figure 21 and Figure 22. At 200 °C, the 2-step extruded $60\left({\mathrm{LDPE}}_{\mathrm{KB}\;16.7\;\mathrm{CNT}\;4.2}\right)/40\mathrm{PBE}$ blends revealed to have higher fluidity than the other blends (Figure 21). The high viscosity of all the blends except the $60\left({\mathrm{LDPE}}_{\mathrm{KB}\;16.7\;\mathrm{CNT}\;4.2}\right)/40\mathrm{PBE}$ one might be explained by the migration of the fillers in the PBE phase. Therefore, it was necessary to increase both the temperature and the weight of the experiment to 245 °C and 10 kg in order to compare the values of the studied polymer nanocomposites (Figure 22). Thus, this temperature was chosen for the 3D printing experiment presented below. 

The extrusion of the nanocomposite blends in two steps led to a more selective location (Figure 22) of the fillers which increased the MFI of the various blends. Indeed, for each blend there is a 20% of the increase in the MFI of the 2-step extrusion compared to the 1-step one. The MFI of the 1-step and 2-step extruded $80\left({\mathrm{LDPE}}_{\mathrm{KB}\;12.5\;\mathrm{CNT}\;3.1}\right)/20\mathrm{PBE}\;$blend is lower than the one of ${\mathrm{LDPE}}_{\mathrm{KB}\;10\;\mathrm{CNT}\;2.5}$**.** This might be due to the higher amount of fillers which highly block the molecular chains of LDPE and PBE if there is a migration of the fillers in the PBE. By increasing the percentage of PBE, there is an increase of the MFI. Indeed, if there is no migration of fillers to PBE in the $60\left({\mathrm{LDPE}}_{\mathrm{KB}\;16.7\;\mathrm{CNT}\;4.2}\right)/40\mathrm{PBE}$ blend, the volume of PBE in the co-continuous biphasic blend is higher which decreases the viscosity of the nanocomposites. However, the MFI of the $60\left({\mathrm{LDPE}}_{\mathrm{KB}B\;16.7\;\mathrm{CNT}\;4.2}\right)/40\mathrm{PBE}\;$remains still much lower than the one of the 60LDPE/40PBE (Figure 4) because of the selective location of KBs and CNTs (Figure 16) which block the molecular chains through Van der Waals forces, and thus, decrease its fluidity. 

#### 3.3.4. Electrical Conductivity 

**Figure 23:**
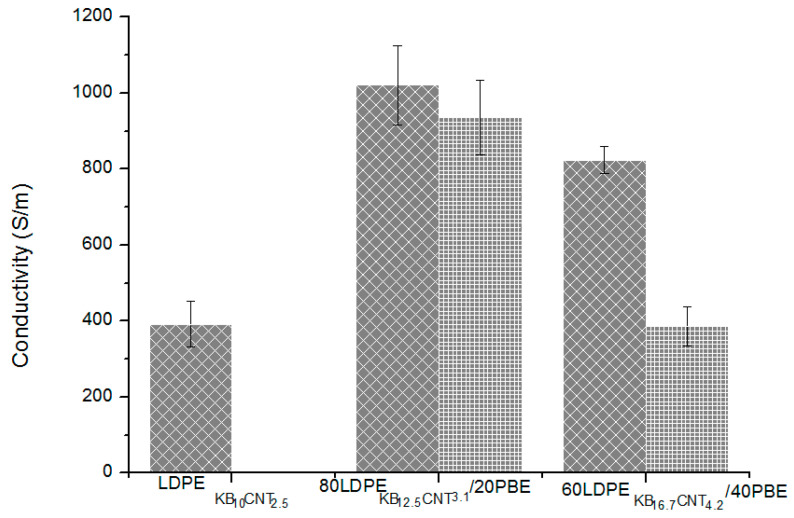
Electrical conductivity (S/m) of the conducting biphasic blends produced by a 1-step (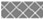)or 2-step (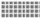) extrusion.

The electrical conductivity of the conducting polymer composites monofilaments was determined and is presented in Figure 23. The global amount of fillers of each blend is 10 wt% of KB and 2.5 wt% of CNT. In the case of addition of 20 wt% or 40 wt% of PBE to conducting LDPE, the electrical conductivity is similar or higher than the one without PBE. Besides, the use of a 1-step extrusion trends to enhance the electrical conductivity compared to the 2-step extrusion one. This might be due to the creation of additional networks between the CNTs and the KBs present in the LDPE, while in the 2-step there is only KB nanoparticles. The dispersion and inter-connections between the fillers also influence the electrical paths and conductivity. 

### 3.4. Properties of 3D-PPOT using Biphasic Polymer Blends

#### 3.4.1. Tensile Properties

**Figure 24:**
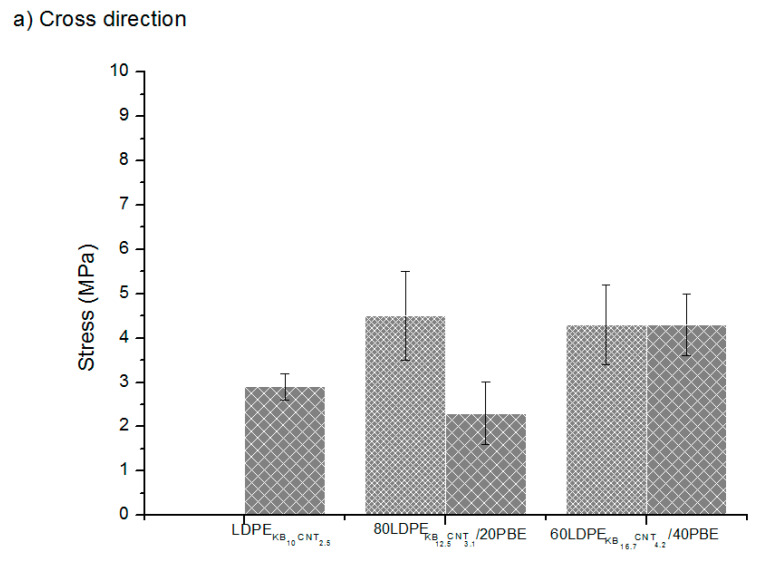

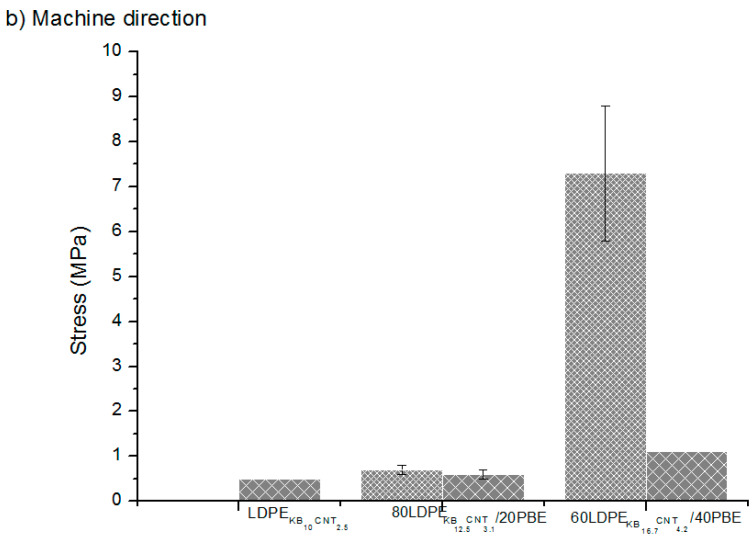
Stress (MPa) of the conducting biphasic blends produced by a 1-step (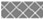) or 2-step (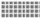) extrusion 3D-printed onto textile materials in the cross (**a**) and machine (**b**) directions of the fabric.

The tensile properties of the conducting nanocomposite polymers of the design of experiment number 2 deposited onto textiles through 3D printing were investigated in the cross and machine directions. Cross and machine directions correspond to the orientation of the fabric for the 3D printing. Eutionnat-Diffo et al. [4] demonstrated that the stress and the strain of the 3D-printed track were lower than the one of the fabric. The stress and strain of the 14 picks/inch PET twill fabric are 18.6 ± 0.2 MPa and 27.9 ± 0.5 % [4]. The influence of the extrusion types on the maximum stress at rupture for various ratios of PBE in the blends (LDPE/PBE) at a similar filler rate was analyzed and is presented in Figure 24. It was shown that all the blends demonstrated similar or higher stresses in both the machine and cross directions. Moreover, in general, the use of a 2-step extrusion enhanced the stress at rupture compared to the 1-step extrusion. The 2-step extruded $60\left({\mathrm{LDPE}}_{\mathrm{KB}\;16.7\;\mathrm{CNT}\;4.2}\right)/40\mathrm{PBE}\;\mathrm{blend}\;$revealed to have the highest stress at rupture. According to Figure 22, the MFI of the $60\left({\mathrm{LDPE}}_{\mathrm{KB}\;16.7\;\mathrm{CNT}\;4.2}\right)/40\mathrm{PBE}\;\mathrm{blend}$ is higher than the other ones which means that this blend is more fluid. Indeed, the change in the filler locations and the second extrusion might have slightly degraded the molecular chains and, therefore, reduced the macromolecular chains leading to a decrease of the viscosity. During the 3D printing process in the machine direction (weft yarn direction), the $60\left({\mathrm{LDPE}}_{\mathrm{KB}\;16.7\;\mathrm{CNT}\;4.2}\right)/40\mathrm{PBE}\;$blend can more easily go through the structure of the textile’s top surface and be entangled to fibers located there. Therefore, the fibers tend to reinforce the tensile properties of 3D-PPOT material using this blend, especially the stress at rupture.

**Figure 25:**
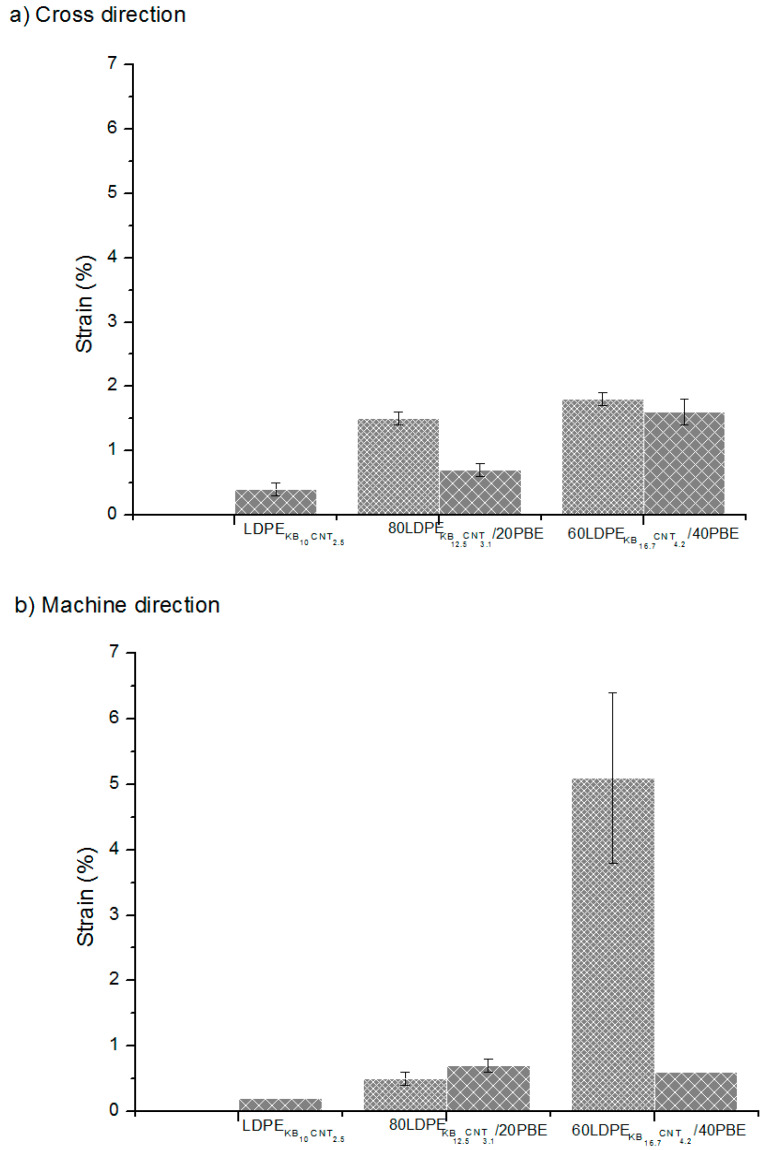
Strain (%) of the conducting biphasic blends produced by a 1-step (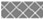) or 2-step (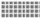) extrusion 3D-printed onto textile materials in the cross (**a**) and machine (**b**) directions of the fabric.

The strain of the conducting polymer composites 3D-printed onto fabrics was improved if they were blended to propylene-based elastomer (PBE) (Figure 25). Indeed, the selective location of the fillers in the thermoplastic phase and at the interface allowed the creation of a highly structured network between the KB and CNT fillers while maintaining the flexibility and elasticity of the PBE phase conferred by its soft segments. 

The 1-step extruded CPCs/PBE blends presented a lower strain at rupture, which might be due to the migration of the fillers to the elastomer’s phase. Indeed, the creation of the new networks reduced the mobility of the macromolecular chains of the soft segments of the PBE, and thus decreased its elasticity.

#### 3.4.2. Deformation Properties 

**Figure 26:**
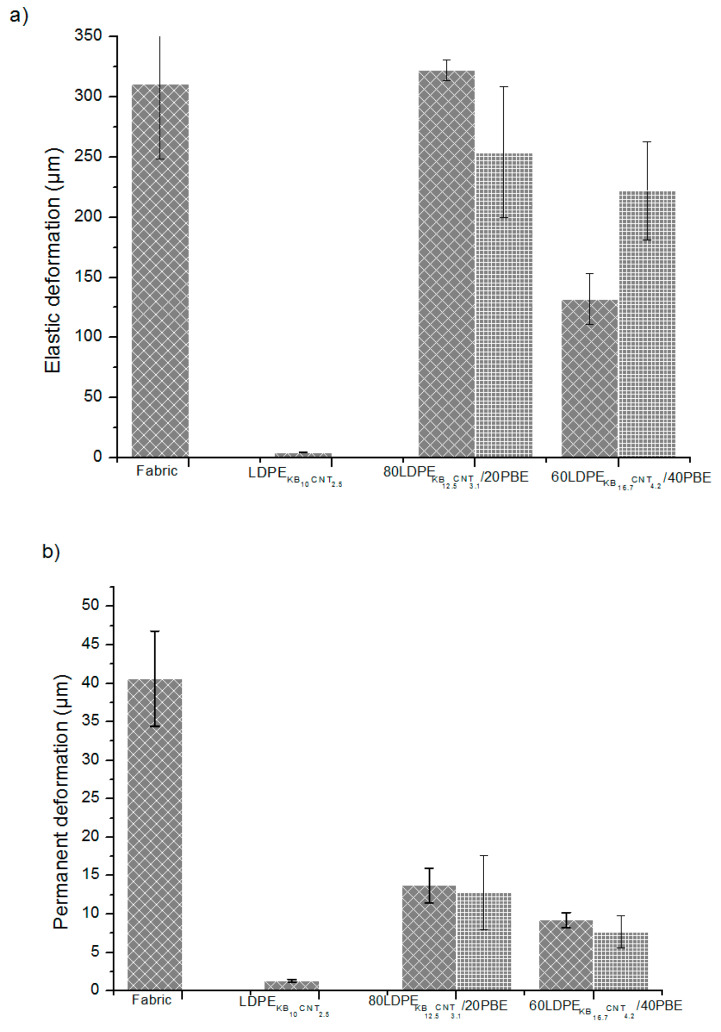
Elastic (**a**) and permanent (**b**) deformations (µm) of the 3D-PPOT materials made of conducting biphasic blends produced by a 1-step (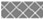) or 2-step (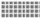) extrusion.

The permanent and elastic deformations of the 3D-printed conducting biphasic blends of the design of experiment 2 were explored (cf. Section 2.2.1). Previously, it was already demonstrated that the permanent and elastic deformations of the conducting polymer composites (PLA + 2.5wt%CB) 3D-printed onto textiles were three times lower than the ones of the textile prior to the 3D printing process [4]. With its low glass temperature, the LDPE is more deformable at ambient temperature than the PLA. However, once the CNT and KB fillers are dispersed in the LDPE matrix, the network created between the fillers avoids the mobility of molecular chains. The addition of PBE in the CNT and KB-filled LDPE composite led to considerably increasing the elastic and permanent deformations of the 3D-PPOT materials up to the value of the original fabric prior to 3D printing (Figure 26). In other words, flexible conducting polymer composites made of the CPCs/PBE biphasic blends for 3D printing onto textiles were successfully developed and demonstrated improved deformability properties. However, the permanent deformation was revealed to be lower than the one of the textile which means that the 3D-PPOT material is more stable. Besides, the higher the percentage of PBE, the higher the elastic deformation and the lower the permanent deformation of the 3D-PPOT materials using CNT and KB-filled LDPE/PBE. Additionally, no significant change between the two modes of extrusion was noticed for the elastic and permanent deformations.

#### 3.4.3. Electrical Conductivity Properties 

**Figure 27:**
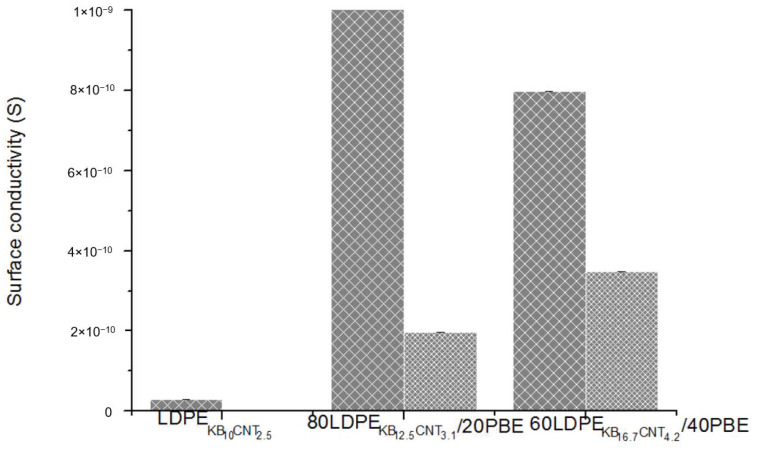
Surface conductivity (S) of the 3D-PPOT materials made of conducting biphasic blends produced by 1-step (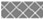) or 2-step (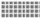) extrusion.

The electrical conductivity of the CNT and KB-filled LDPE/PBE biphasic blend 3D-printed onto textiles was measured and reported in Figure 27. Similar trends of results are observed between the electrical conductivity of the monofilaments and the 3D-PPOT materials. In the case of addition of 20% or 40% of PBE to conducting LDPE, the electrical conductivity of the 3D-PPOT is higher than the one without PBE. Moreover, the use of a 1-step extrusion trends to enhance the electrical conductivity compared to the 2-step extrusion one. In other words, the 3D printing might not influence the dispersion of the fillers in the various blends. 

## 4. Conclusions

In this study, flexible and conductive monofilaments for functional textiles through 3D printing of biphasic polymer blends onto textiles were developed and characterized. Indeed, immiscible carbon nanotubes (CNT) and Ketjenblack (KB)-filled low-density polyethylene (LDPE)/propylene-based elastomer (PBE) blends at different ratios were created in order to enhance the flexibility, stress and strain at rupture and electrical conductivity of these conductive polymer composites 3D-printed onto textiles. 

The important requirements to improve these properties were the co-continuity of both the thermoplastic and the elastomer phases and also the location of the conductive fillers in the thermoplastic phase or at the interface of the two immiscible polymers. Indeed, the selective location of the fillers in the thermoplastic phase allowed the creation of a highly structured network between the KB and CNT fillers while maintaining the flexibility and elasticity of the PBE phase conferred by its soft segments. 

The findings demonstrate that in case of the use of a two-step melt processing, the CNT and KB particles are found to be preferentially located at the LDPE/PBE interface and in the LDPE phase and at the LDPE/PBE interface, respectively. These selective locations support the extensions of the co-continuity of the LDPE and PBE phases over a much larger composition range. Therefore, the viscosity and electrical conductivity of monofilament, the deformation under compression, the strain and stress and the electrical conductivity of the 3D-printed conducting polymer composite onto textiles were significantly improved with KB and CNT-filled LDPE/PBE blends compared to KB and CNT-filled LDPE. 

The two-step extrusion processing of the $60\left({\mathrm{LDPE}}_{\mathrm{KB}\;16.7\;\mathrm{CNT}\;4.2}\right)/40\mathrm{PBE}$ blends presented great properties almost similar to the ones of the textile materials and, henceforth, could be a better material for functional textile development through 3D printing onto textiles. However, compromises should be found in order to develop these textiles with high conductivity as well as high flexibility and mechanical properties

## Figures and Tables

**Table 1:** Sample descriptions of the first design of experiment.

Sample Reference	Composition of (KB and CNT)-Filled LDPE *			Composition of KB- and CNT-Filled LDPE/PBE Blends			
	wt% KB	wt% CNT	wt% LDPE	wt% (KB and CNT)-Filled LDPE			wt% PBE
				wt% KB	wt% CNT	wt% LDPE	
**A1**	0	0	100	0	0	60	40
**A2**	5	0	95	5	0	95	0
**A3**	10	0	90	10	0	90	0
**A4**	10	2.5	87.5	10	2.5	87.5	0
**A5**	10	5	85	10	5	85	0
**A6**	5	0	95	3	0	57	40
**A7**	10	0	90	6	0	54	40
**A8**	10	2.5	87.5	6	1.5	52.5	40
**A9**	10	5	85	6	3	51	40
**A10**	5	0	95	2	0	38	60
**A11**	10	0	90	4	0	36	60
**A12**	10	2.5	87.5	4	1	35	60
**A13**	10	5	85	4	2	34	60
**A14**	0	0	100	0	0	40	60

* (KB and CNT)-filled LDPE means low-density polyethylene (LDPE) filled with Ketjenblack (KB) and carbon nanotube (CNT) nanoparticles.

**Table 2:** Sample descriptions of the second design of experiments.

Sample Reference	Composition of KB- and CNT- Filled LDPE Formulations			Composition of KB- and CNT-Filled LDPE/PBE Blend Formulations				Extrusion Scenario
	wt% KB	wt% CNT	wt% LDPE	wt% (KB and CNT)- Filled LDPE			wt% PBE	
				wt% KB	wt% CNT	wt% LDPE		
**B1**	10	2.5	87.5	10	2.5	87.5	0	1-step ^1^
**B2**	12.5	3.1	84.4	10	2.5	67.5	20	1-step
**B3**	16.7	4.2	79.1	10	2.5	47.5	40	1-step
**B4**	12.5	3.1	84.4	10	2.5	67.5	20	2-step ^2^
**B5**	16.7	4.2	79.1	10	2.5	47.5	40	2-step

^1^ Dispersion of the KB and CNT in LDPE and propylene-based elastomer (PBE) in one step (see Figure 1). ^2^ Dispersion of the KB and CNT in LDPE and then blended with PBE (Figure 1).

**Table 3:** Temperature profiles (°C) of the extrusion of the LDPE-based CPCs and polymer blends.

Sample Reference ^1^	Description of Polymer Blends	T_1_ (°C)	T_2_ (°C)	T_3_ (°C)	T_4_ (°C)	T_5_ (°C)
A5 A9 A13	${\mathrm{LDPE}}_{\mathrm{KB}\;10\;\mathrm{CNT}\;5}$ $40{\mathrm{LDPE}}_{\mathrm{KB}\;10\;\mathrm{CNT}\;5}/60\mathrm{PBE}$ $60{\mathrm{LDPE}}_{\mathrm{KB}\;10\;\mathrm{CNT}\;5}/40\mathrm{PBE}$	125	175	215	225	240
A4, B1 A8 A12	${\mathrm{LDPE}}_{\mathrm{KB}\;10\;\mathrm{CNT}\;2,5}$ $40{\mathrm{LDPE}}_{\mathrm{KB}\;10\;\mathrm{CNT}\;2,5}/60\mathrm{PBE}$ $60{\mathrm{LDPE}}_{\mathrm{KB}\;10\;\mathrm{CNT}\;2,5}/40\mathrm{PBE}$	125	175	210	220	240
A3 A7 A11	${\mathrm{LDPE}}_{KB\;10\;}$ $40{\mathrm{LDPE}}_{\mathrm{KB}\;10\;}/60\mathrm{PBE}$ $60{\mathrm{LDPE}}_{\mathrm{KB}\;10\;}/40\mathrm{PBE}$	125	175	205	215	240
A2 A6 A10	${\mathrm{LDPE}}_{\mathrm{KB}\;5\;}$ $40{\mathrm{LDPE}}_{\mathrm{KB}\;5\;}/60\mathrm{PBE}$ $60{\mathrm{LDPE}}_{\mathrm{KB}\;5\;}/40\mathrm{PBE}$	125	175	195	210	230
A14	40LDPE/60PBE	125	170	175	180	200
A1	60LDPE/40PBE	125	170	175	180	200
B3, B5	$60{\mathrm{LDPE}}_{\mathrm{KB}\;16,7\;\mathrm{CNT}\;4,2\;}/40\mathrm{PBE}$	120	220	250	260	270
B2, B4	$80{\mathrm{LDPE}}_{\mathrm{KB}\;12.5\;\mathrm{CNT}\;3,1\;}/20\mathrm{PBE}$	120	220	250	260	270

^1^ Sample reference of first (sample A1–A14) and second design (sample B1–B5) of experiments already described in Table 1; Table 2.

**Table 4:** 3D printing parameters.

Parameters	Values (Unit)
**Infill percentage**	100 (%)
**Z offset (distance between the head)**	0 (mm)
**Printing speed**	3600 (mm/mi)
**Extruder diameter**	0.4 (mm)
**Extruder temperature**	245 (°C)

**Table 5:** Surface tensions of water and α-bromonaphthalene.

Liquid	γL (mN/m)	γL^D^ (mN/m)	γL^P^ (mN/m)
Water	72.6	21.6	51
α-bromonaphthalene	44.6	44.6	0

**Table 6:** Contact angle (°) of PBE with water and α-Bromonaphtalene.

	Contact Angle (°)	
	Water	α-Bromonaphtalene
**PBE**	102.1 ± 1	45.6 ± 2

**Table 7:** Surface tensions (mN/m) of LDPE, PBE, CNT and KB at room temperature.

Material	Y_s_ (mN/m)	Y_s_^d^ (mN/m)	Y_s_^p^ (mN/m)
**LDPE**	33.2 [36]	33.2 [36]	0 [36]
**PBE**	11.7	5.5	6.3
**CNT**	27.8 [19]	17.6 [19]	10.2 [19]
**KB (carbon black)**	71.2 [37]	36.8 [37]	34.4 [37]

**Table 8:** Wettability coefficient and predicted location of fillers in conducting LDPE/PBE blends.

	KB	CNT
**ω _PBE / LDPE_**	0.31 ± 0.02	−0.29 ± 0.01
**Location of fillers**	Interface	Interface
